# Abdominal Cystotomy

**Published:** 1900-12-08

**Authors:** 


### ABDOMINAL CYSTOTOMY.

At the last meeting of the Royal Medical and Chirurgical Society Sir Thomas Smith raised the question whether in the operation of supra-pubic cystotomy for the removal of stones or morbid growths from the bladder it might not be possible to improve the present method of procedure. According to current methods the aim is to get into the bladder without opening the peritoneal cavity; but in view of the greatly diminished risk which now attends interference with the peritoneum Sir Thomas Smith suggested that in cases where it was desirable to obtain immediate union of the bladder wound it might be worth while to make the operation intra-peritoneal, so as to take advantage of the facility with which opposed surfaces of peritoneum unite after suture. If such a thing were done it would be desirable to adopt the Trendelenburg position, to distend the bladder with air rather than with fluid, to open the bladder at a higher point than that usually selected, and of course to close the bladder by immediate suture. In the discussion which followed the only serious objection raised to the suggested proceeding was that wherever such an operation was likely to be required the bladder was almost sure to be septic, and that in such cases the risk of infecting the peritoneum was one which should not be incurred. As Mr. Christopher Heath put it "it was flying in the face of providence to open the peritoneum when it could be done without."

Other speakers, however, seemed to think that too much had been said about the risks of sepsis, and that they could be avoided by washing out the bladder before the operation, a suggestion as to the feasibility of which we fancy there will be two opinions.

Mr. W. G. Spencer, who supported the views of Sir Thomas Smith, very properly pointed out that the suggested operation should be regarded in every way as an abdominal one, and not as a mere extension or modification of supra-pubic lithotomy.

